# Supplementary material for: Creating frames of reference for chert exploitation during the Late Pleistocene in Southwesternmost Iberia
Source: PLoS One. 2023 Oct 20;18(10):e0293223. doi: 10.1371/journal.pone.0293223 (PMC10588902; doi:10.1371/journal.pone.0293223)
Supplement: S3 Table — Description of the used variables (including allowed variables and references) for the macroscopic and petrographic analyses used in the present study. (PDF) [file pone.0293223.s003.pdf]

Data dictionaries for macroscopic and petrographic analysis

Data dictionary for macroscopic analysis

Data dictionary for macroscopic description of the geological samples.

Variable	Allowed values	Description	Reference
Color	-	Color according to the Munsell Soil Color Chart.	
Fabric	Homogeneous, Heterogeneous	Distribution of color, luster, translucency, feel and pattern in the sample. Heterogeneous is the presence of different characteristics within one single sample.	According to Luedtke (1992).
Luster	Shiny, Medium, Dull.	The character of light reflected by minerals. Shiny refers to when the piece has a very intense luster; Medium refers to when the piece has some luster but it is not very intense; Dull refers to when the piece has no luster.	According to Crandell (2005) and Luedtke (1992).
Translucency - qualitative	Highly translucent, Translucent, Sub-translucent, Opaque.	Degree to which the light can penetrate a material when held 5cm from a 40w lamp. Highly translucent - transparent or near transparent; Translucent - silhouettes can be seen through the sample's thin part and/or light passes through the thick parts; Sub-translucent - light only passes through the thin parts; Opaque - no or almost no light passes through.	According to Bressy (2002) and Crandell (2005).
Feel	Smooth, Semi-smooth, Rough.	A fingernail is dragged across the sample's surface. Smooth - nothing can be felt; Semi-smooth - slight rough feel; Rough - if the sample is distinctly rough.	According to Bressy (2002) and Crandell (2005).
Pattern	Shaded, Spotted, Splotched, Broad mottling, Marbled	Type of pattern present on the sample. Spotted - circle patterns less than 30% of the surface area; Splotched -	According to Crandell (2005).

	mottling, Speckling, Flecks, Banded, Streaked, Laminated, Finely laminated.	irregular shapes less than 30% of the surface area; Broad mottling - large, irregular blotching, covering more than 30% of the surface; Marbled mottling - large relatively round shapes, covering more than 30% of the surface; Speckling - small dots, well distributed over the surface; Flecks - small dots, grouped together.	
Cortex	Dolomitic, Limestone, Unknown.	Acid test to obtain chemical nature. A drop of 2% HCI solution is deposited on fresh cortex surface to discriminate between carbonate and siliceous nature. Application to archaeological materials will be limited.	According to Delluniversità et al (2019).
Fracture	Conchoidal, Uneven.	Presence or absence of a conchoidal fracture.	
Quality	Good, Acceptable, Low.	Combination of: 1) homogeneity, understood as the lack of fractures, flaws and cracks; 2) fine granularity and smooth feel; 3) lack of inclusions that due to their nature or distribution impact the knapping.	According to Luedtke (1992), Brandl (2013).

Data dictionary for petrographic analysis

Data dictionary for petrographic description of the geological samples.

Variable	Allowed values	Description	Reference
ID		Individual thinsection ID.	
Outcrop/level		Outcrop name or archaeological level.	
Lithology		Type of rock	
Texture	Mudstone, Wackestone, Packstone, Grainstone, Boundstone, Other	Mudstone: Muddy carbonate rock containing less then 10 % grains; Wackestone: Mud-supported carbonate rock containing more than 10 % grains; Packstone: Grain-supported muddy carbonate rock; Grainstone: Mud-free carbonate rocks, which are grain supported; Boundstone: Carbonate rocks showing signs of being bound during deposition.	According to Dunham (1962)
Microstructure	Homogeneous, Banded, Laminar, Nodular, Brexoid, Other	Distribution of crystals and clasts within the rock at a microscopic scale. Homogeneous: equally spread in the rock; Banded: distributed in bands; Nodular: distributed in clumps; Brechoid: fracturing of the rock irregularly.	According to Dorado (1989, pp. 21)
Orthochem		Materials formed in two ways: 1) deposited directly from supersaturated aqueous solutions due to chemical reactions or evaporation; 2) formed by the replacement of existing sedimentary materials.	According to Vernon (2018, pp. 24-25)
Orthochem type	Essential (ES), Accessory (AC), Secondary (SE)	Essential: minerals that form more than 5% of the volume of the rock; Accessory: Minerals with proportion of less than 5% of the volume of the rock; Secondary: products of the alteration (hydrothermal or physical), independent of the proportion within the rock.	According to Dorado (1989, pp. 26)
Orthochem		General description of the orthochem and where it is	

description		identified.	
Orthochem (%)		Approximate percentage of the orthochem's presence in the total thin-section area.	
Allochem		Material formed by the movement and reorganization into new shapes by chemical, physical or biological processes within the depositional basin (ex. ooliths, fecal pellets, iron oxide minerals).	According to Vernon (2018, pp. 25, 27)
Allochem (freq)	Rare, Uncommon, Common, Very frequent	Rare: present one or two elements; Uncommon: present three to 10 elements; Common: present 11 to 20 elements; Very frequent:> 20 elements.	
Bioclast		Also known as skeletal particles, are the remains (complete or fragmented) of the hard parts of carbonate-secreeting organisms.	According to Adams, McKenzie and Guilford (1991, pp. 39)
Bioclast (freq)	Rare, Uncommon, Common, Very frequent	Rare: present one or two elements; Uncommon: present three to 10 elements; Common: present 11 to 20 elements; Very frequent:> 20 elements.	
Porosity (%)		Approximate frequency of effective porosity.	
Porosity type	Interparticle, Moldic, Fenestral, Fracture, Vuggy, Shelter, Other	Interparticle: porosity between particles; Moldic: Porosity formed by selective removal of an individual constituent of the rock; Fenestral: Pores larger than grain-supported interstices (interparticle); Fracture: Porosity formed by fracturing; Vug: Pores larger then 1/16 mm in diameter and somewhat equant in shape; Shelter: Porosity created by the sheltering effect of large sedimentary particles; Other: Other types of porosity.	According to Choquette and Pray (1970)
Sedimentary	Parallel lamination, Convoluted lamination,	Parallel lamination: sedimentary strata less than 10 mm thick, recognizable due to variation in structure or	According to Middleton et

structures	Bands/zonations, Burrow, Other	composition and more or less parallel bounding surfaces; Convoluted lamination: symmetrical about a vertical plane or leaning and asymmetrical, and usually exhibit narrow vertical upturned laminae, often truncated at the top, separated by a broader synclinal downfolds; Bands/zonations: limited areas with different characteristics related to changes in the sedimentation or cementation process; Burrow: bioturbation structures caused by activity of an organism that disrupts the stratification features; Other: Other types of sedimentary structures.	al. (2003)

References

1. Luedtke BE. An Archaeologist's Guide to Chert and Flint. 1992.

2. Crandell O. Macroscopic Analysis and Characterisation of Chert for Provenance Purposes. Sargetia, Acta Musei Devensis. 2005;33: 137–153.

3. Bressy C. Caracterisation et gestion du silex des sites mesolithiques et neolithiques du Nord-Ouest de L'Arc Alpin. Une approche pétrographique et géochimique. Doctoral thesis, Université de Aix-Marseille. 2002.

4. Delluniversità E, Muntoni IM, Allegretta I, Tarantini M, Monno A, Maiorano P, et al. Development of a multiparametric characterisation protocol for chert investigation and application on the Gargano Promontory mines. Archaeol Anthropol Sci. 2019;11: 6037–6063. doi:10.1007/s12520-019-00875-8

5. Brandl M. Genesis, Provenance and Classification of Rocks within the Chert Group in Central Europe. Archaeologia Austriaca. 2013;97/98: 33–58.

6. Middleton V. Encyclopedia of Sediments and Sedimentary Rocks. Springer Netherlands; 2005.

7. Dunham R.J. Classification of Carbonate Rocks According to Depositional Texture. In: Ham, W.E., Ed., Classification of Carbonate Rocks, AAPG, Tulsa, 1962:108-121.

8. Castro Dorado A. Petrografía básica: texturas, clasificación y nomenclatura de rocas. Madrid: Paraninfo; 1989.

9. Vernon RH. Microstructures of Sedimentary Rocks. A Practical Guide to Rock Microstructure. 2nd ed. Cambridge: Cambridge University Press; 2018. pp. 7–27. doi:10.1017/9781108654609.004

10. Adams, A., MacKenzie, W., Guilford, C. Atlas of sedimentary rocks under the microscope. 1991.

11. Choquette P., Pray L. Geologic Nomenclature and Classification of Porosity in Sedimentary Carbonates. Bulletin. 1970;54. doi:10.1306/5D25C98B-16C1-11D7-8645000102C1865D
