# Supplementary material for: Creating frames of reference for chert exploitation during the Late Pleistocene in Southwesternmost Iberia
Source: PLoS One. 2023 Oct 20;18(10):e0293223. doi: 10.1371/journal.pone.0293223 (PMC10588902; doi:10.1371/journal.pone.0293223)
Supplement: Attachment — Submitted filename: Response to reviewers.docx [file pone.0293223.s005.docx]

Reviewer #2:

Reviewer #2: This is my second review of the article led by Joana Belmiro et al. entitled “Creating frames of reference for chert exploitation during the Late Pleistocene in Southwesternmost Iberia”. After this revision, I can say that the article is definitely improved and several big issues I had with the previous version have been rectified, which I think definitely benefit the paper. However, as the article is intended to be a framework for future studies of raw materials applied to various archaeological sites in Portugal, some more precise data has to be included. I refer mainly to the geological formations with chert and their reflection in the manuscript, especially in figure 3. For example, in the case of this figure, it has been modified and improved, but since throughout the manuscript the geological formations with chert are mentioned, I think that a more detailed map including the geological formations would be necessary, and not just the series/epoch. I believe this is one of the keys of the manuscript to be a solid framework of reference on chert raw materials for further studies applied to archaeological sites. Nevertheless, the study is a definite improvement. We thank reviewer #2 for their suggestions and comments since they provide valuable feedback and have helped improve this revised version of the article. We believe a map showing the geological formations referenced in the paper is an improvement. We created a new map, showing the chert-bearing formations (not just the series/epoch), with the numbered outcrops where chert can be found within the formation.

Regarding the general aspects I now agree with the comments of the authors to continue with the geographic division of western and eastern areas, but I advise to reinforce the idea in geological settings. Just add a sentence mentioning the use of this geographical division because of, as the authors rightly say, the differences in cherts and their formations. A sentence was added to reinforce the geographic division of western and eastern areas in the geological setting.

Still on the same topic "frame of reference", although figure 2 has been improved and enlarged, as there are four chert formations, at least one photograph of each formation should be included. Some pictures of chert from Kimmeridgian formations? Two photos of the Kimmeridgian formation outcrop and cherts have been added to the set, to represent all the chert-bearing formations in the Algarve.

Minor issues:

Line 334. When table S3 is cited, it should not be S2, in order to follow an order. We have corrected the SOM files names, to better reflect their reference order. In this case, the Macroscopic analysis dataset is now S2, and the Data dictionaries are now S3.

Line 366. At this point, here table S2 should be S3. Same as previous commentary.

Line 378. Maybe it's me that's not working but I can't find the DOI in question. The short form of the DOI was replaced by the full link “<https://doi.org/10.17605/OSF.IO/FP7TA>”, which takes the reader to the correct OSF page.

Line 385. Results. Personally, I think there is still a bit of confusion between geological formation with chert, type outcrop, outcrop and sample. Although figure 3 and table 2 has clarified the data much more, looking at the detailed scale of the outcrops it sounds a bit strange to me that an outcrop, not a formation, has cherts covering a distance of almost 10 km. Wouldn't it be better to have the formation well defined and to have as many outcrops as points where cherts are found in the formation and that each point would take the nearby toponym to define the outcrop/location? This is in line with the general comment at the beginning that the delineation of chert formations greatly strengthens this work as a frame of reference. We have clarified the distinction between geological formations with cherts and outcrops throughout the manuscript. The outcrops take the nearby toponym (in some cases, the toponym is the name of the formation, due to it being the name of the area, for example JOR and MALH toponyms), but we have clarified the outcrops to be points where chert is found within the formation.

It would also be helpful to have the outcrops mentioned in the text with their number and acronym before the figure. This would make it easier to follow the manuscript for those of us who are not familiar with the region. Added missing acronyms to the outcrops mentioned in the text, especially before Figure 3 to aid in the comprehension of the location of the outcrops.
